# Answering the question: are we making a difference?

**DOI:** 10.1186/1471-2458-13-S2-S7

**Published:** 2013-06-17

**Authors:** Dale Huntington

**Affiliations:** 1 Asia Pacific Observatory on Health Systems and Policies, World Health Organization, Western Pacific Regional Office, P.O. Box 2932, 1000 Manila, Philippines

## 

Performance-based payment schemes are nothing new for not-for-profit organizations that use commercial business models, such as social marketing and social franchise programs. Perhaps because of their familiarity with using output-based budgeting processes, these programs have advanced the development of different types of modeling techniques for describing impacts on health outcomes, and applied these estimates to monitor programs worldwide. Tried and true estimates of health status have been used in new ways, and innovative estimates of equity are reported. Each of these developments is discussed in this special supplement, and each has something to say about the metrics that are used, and the usefulness of the results they produce.

The disability-adjusted life year metric (DALY) is a well-known measure of a population's disease burden, most commonly known for its use in estimating the global burden of disease. The construction of the DALY statistic has not been without controversy, particularly the relative valuation of some interventions over others and the specification of the weights that are assigned for the measurement of health losses. The Global Burden of Disease Study 2010 provided fresh re-estimations of these weights using large-scale, population-based data sources, representing a major advancement with the DALY metric [1,2]. The variation of the DALY metric to suggest the effectiveness of programs, the DALYs averted metric, is an attractive theoretical premise that several of the papers in this special supplement explore. Through the use of large-scale program performance data across multiple countries, these papers carefully develop original applications of the DALYs averted metric, tracking impact of a single program, and comparing programs across widely different settings. In the process, several interesting issues arise that are surely causing conundrums for managers. For example, the shift from counting the number of products sold to estimating health impact showed that it is the level of disease prevalence (and mortality rates) that will drive the DALYs averted count; smaller programs in high disease burden settings have a larger impact in terms of DALYs averted than large programs in settings with low disease prevalence [3,4].

There are a number of conceptual leaps made in the models being reported, particularly as they move from using products and service utilization statistics to estimating the numbers of DALYs averted based upon the predicted impact of behavior change interventions. The application of these analytic methods across a wide range of services and products within the framework of routine monitoring are innovative achievements, and represent advances to the field of measuring health impact. However, underlying the models are the ubiquitous problems with data quality - even in closely managed social marketing and franchise programs. The broader use of modeling DALYs averted counts will be challenging in settings where the availability of reliable and valid data has been a constraint to capturing accurate measures of performance. The modeling of DALYs averted is a leap in analytic sophistication for programs accustomed to reporting tallies of outputs, as well as more standard rates or ratios as measures of coverage and impact. The requisite skills in complex statistical modeling will be a constraint to the wide-scale diffusion of the DALYs averted technique for monitoring impact.

An alternative approach to measuring a program's impact is to assess its contribution to national health goals, rather than suggest attribution to changes in population health status. Family planning programs are assessed on an aggregate level by changes in the population's contraceptive prevalence and on a programmatic level by couple-years of protection (CYPs). CYPs convert the number of contraceptives distributed into numbers of contraceptive users. The Marie Stopes International Impact 2 model goes the next step by using an innovative technique that estimates a service delivery organization's marginal contribution to population-level contraceptive prevalence rates (CPR) that is made by the number of contraceptive users served (which is derived from the number of products distributed). It is quite a stretch, but the analysis is elegant and the solutions to working around substitution effects are innovative, despite the several underlying assumptions and limitations imposed by the type of data that is used. The results are immediately relevant for national health policy and contribute to making the case for public-private partnership in achieving national health goals. In general, our understanding of the private health sector's expansion far outweighs information about its actual contribution to achieving national health goals. In part, this can be attributed to weak incentives for private sector networks to report utilization statistics to government. Increased understanding of the relative contribution made by private sector organizations will facilitate government regulatory oversight and strengthen public-private partnership linkages. The analytic approach discussed in this supplement is a step in the right direction - let's look for more such evidence as large-scale, not-for-profit programs continue to mature in their operations and relations with government.

Programs that are designed to reach the poor with subsidized services have historically experienced crowding out by the less poor, who are better able to overcome non-financial barriers to access. Ensuring that target populations are reached is an important function of impact metrics, particularly for social marketing and social franchise programs. Wealth quintiles have been an easily recognized and interpretable metric of equity. The concentration index is another measure that has the benefit of being comparable across different settings and times, as well as the drawback of being somewhat difficult to interpret. Additionally, the concentration index is comparable only to itself, thereby making it less useful in sectoral policy dialogue. A practical solution presented in in this supplement by Chakraborty et al. combines the two metrics and incorporates a national reference population for comparative analyses [5]. The results showed that social marketing programs were reaching groups that were wealthier than the national samples, tending towards the higher quintiles rather than the poorer. This challenges the notion that social marketing programs reach a different group than commercial marketing. As this finding goes to the heart of a basic strategy behind the social marketing concept, further evidence is needed to determine if the crowding out phenomenon is taking place and, if so, why. The bottom-line indicator should not be lost in this discussion, however: Does the social marketing program increase utilization or does it simply cause a shift in sources of care among those who are presently covered by the national health program? Government may be more effective in reaching the poor, and the private sector more effective in growing coverage among the near poor and middle class. This is not necessarily the strategy of social marketing programs, but it would resonate in many policy discussions.

Taken as a whole, the papers in this special issue convey insights into the extensive monitoring and evaluation of two of the large, not-for-profit global health programs. Substantial resources are being used to convert the measures of services delivered and commercial products sold or distributed that were being routinely collected by social marketing and social franchise programs as output metrics into something more multi-dimensional to suggest impact. This is nicely done with rigor and sophistication. Where gaps exist between the data and theory, informed assumptions are made, proxies are used, and data are run through the models. This modeling should encourage other service delivery organizations to move beyond performance measures and to create models of impact. Strategic decisions on how to assess impact will need to be made, however, balancing investments in upgrading analytic capacity using routinely collected statistics versus mounting special studies that utilize primary data collection methods. Final decisions on how any given program, or organization can best report on its impact will depend on a mix of factors. The importance of moving beyond measures of performance to impact is well understood and, as the papers in this special edition reveal, innovative techniques are being developed to respond to the simple question, "Are we making a difference?"

## List of abbreviations

DALY: disability-adjusted life year; CYPs: couple-years of protection; CPR: contraceptive prevalence rate

## Competing interests

The author declares that he has no competing interests.

## Declarations

This article is part of the supplement of *BMC Public Health *Volume 13, Supplement 2, 2013: Use of health impact metrics for programmatic decision making in global health. Population Services International, a registered non-profit organization, provided the funding for the publication of this supplement. The full contents of the supplement are available online at http://www.biomedcentral.com/bmcpublichealth/supplements/13/S2.
